# Oculomotor phenotypes and longitudinal video-oculography dynamics in anti-GAD65 antibody–associated neurological syndromes: a case series and literature review

**DOI:** 10.1186/s12883-026-05014-0

**Published:** 2026-06-11

**Authors:** Feifei Dai, Shilei Cui, Yanjun Guo, Jiawei Wang

**Affiliations:** Neurology Department of Beijing Tongren Hospital, Capital Medical University, Beijing, China

**Keywords:** GAD65, Ocular manifestations, Vestibulo-ocular motor, Video-oculography, Titin

## Abstract

**Background:**

Anti–glutamic acid decarboxylase (GAD) antibody–associated neurological syndromes encompass a heterogeneous group of immune-mediated disorders in which ocular involvement is increasingly recognized but remains insufficiently characterized. Data on clinical features, neuroimaging, and longitudinal ocular motor findings are limited.

**Methods:**

We retrospectively reviewed four patients diagnosed with anti-GAD antibody–associated neurological syndromes presenting with ocular manifestations at our center between 2019 and 2025. Clinical features, ocular motor findings, laboratory and neuroimaging results, treatment, and outcomes were collected. Longitudinal video-oculography (VOG) was analyzed in one patient. A literature review was performed to summarize reported ocular and vestibulo-ocular abnormalities.

**Results:**

All four patients (2 males, 2 females; onset age 53–68 years) exhibited ocular symptoms, three presented with ocular symptoms early in the disease course; however, in one case (Case 4), the temporal association between the initial ocular symptom and the final diagnosis remained uncertain. Diplopia, nystagmus, and ptosis were the predominant findings. Early brain and orbital Magnetic Resonance Imaging (MRI) were unremarkable, while cerebellar atrophy developed in one patient during relapse. All patients were positive for serum anti-GAD65 antibodies; one also had anti-titin antibodies with elevated creatine kinase(CK). VOG in one patient demonstrated worsening saccadic velocity and pursuit gain during relapse, consistent with cerebellar dysfunction. Literature review of 61 articles showed that diplopia and nystagmus were the most frequently reported features, with characteristic vestibulo-ocular abnormalities including multistep saccades and square-wave oscillations. Based on clinical and literature findings, we further identified recurrent oculomotor patterns across cases.

**Conclusions:**

Ocular symptoms may represent early clinical features of anti-GAD antibody–associated neurological syndromes, although the temporal relationship may vary across patients and should be interpreted with caution in atypical cases. Downbeat nystagmus may serve as an early marker of cerebellar involvement. Longitudinal VOG provides objective insights into disease evolution. Importantly, we propose a structured oculomotor phenotype framework and highlight longitudinal VOG as a tool for tracking dynamic cerebellar dysfunction, which may facilitate earlier recognition and monitoring of disease progression.

**Supplementary Information:**

The online version contains supplementary material available at 10.1186/s12883-026-05014-0.

## Introduction

Anti–glutamic acid decarboxylase (GAD) antibody–associated neurological syndromes comprise a broad spectrum of immune-mediated disorders, including stiff-person syndrome, cerebellar ataxia, limbic encephalitis, and epilepsy [1–4]. Among these conditions, ocular involvement has been increasingly recognized but remains relatively uncommon. Reported manifestations include nystagmus, diplopia, ptosis, ocular flutter, opsoclonus, and various saccadic abnormalities [5–12], reflecting potential cerebellar, brainstem, or ocular motor pathway dysfunction. However, the clinical characteristics and diagnostic implications of these ocular findings are not well defined.

Evidence describing detailed ocular phenotypes, longitudinal evolution of ocular motor dysfunction, and correlations with neuroimaging or autoimmune profiles is limited. In particular, few studies have incorporated video-oculography (VOG) to objectively document dynamic changes over the disease course. Furthermore, atypical presentations—such as coexisting antibodies or muscle involvement—are rarely reported, leaving clinicians with limited guidance when evaluating patients presenting primarily with ocular symptoms.

In this study, we describe four patients with anti-GAD antibody–associated neurological syndromes presenting with prominent ocular manifestations and analyze their clinical, laboratory, neuroimaging, and longitudinal VOG features. We also summarize published ocular and vestibulo-ocular findings through a comprehensive literature review. Our aim is to improve early recognition of ocular presentations and to highlight the diagnostic value of ocular motor assessment in this patient population.

## Methods

### Study design and patients

We conducted a retrospective observational study of patients diagnosed with anti–glutamic acid decarboxylase (GAD) antibody–associated neurological syndromes who presented with ocular manifestations at our center between January 2019 and December 2025. Inclusion criteria were: (1) positivity for serum anti-GAD65 antibodies, (2) presence of ocular symptoms, and (3) accompanying neurological signs. Patients with cerebrovascular disease were excluded. Demographic information, clinical presentations, disease course, treatment, and outcomes were collected from medical records. Written informed consent was obtained from all patients or their legal guardians.

### Clinical assessment and cognitive assessments

All patients underwent standardized neurological and ophthalmological evaluation by board-certified neurologists and neuro-ophthalmologists. Ocular assessments included evaluations of diplopia, ptosis, nystagmus, gaze limitation, saccadic function, and smooth pursuit. Neurological disability was quantified using the modified Rankin Scale (mRS) [13], a widely used measure of global disability after neurological disease, at onset, maximal severity, and last follow-up. Cognitive performance was assessed using the Montreal Cognitive Assessment (MoCA) [14], a screening tool designed to detect mild cognitive impairment. Detailed case timelines are provided in Supplementary Table S1.

### Laboratory testing

Routine laboratory studies included complete blood count, metabolic panel, coagulation profile, erythrocyte sedimentation rate, and serum tumor markers. Autoimmune screening comprised antinuclear antibodies (ANA), extractable nuclear antigen (ENA) antibodies, anti–double-stranded DNA antibodies, antineutrophil cytoplasmic antibodies, anticardiolipin antibodies, rheumatoid factor, angiotensin-converting enzyme, and HLA-B27(human leukocyte antigen-B27).All patients were screened for diabetes mellitus and thyroid dysfunction. Cerebrospinal fluid (CSF) analysis was performed when clinically indicated. Autoimmune and paraneoplastic antibody panels were assessed in serum and/or CSF using semi-quantitative immunoblot and expressed as dilution ratios, including antibodies against GAD65, NMDA receptor, AMPA receptor, GABAB receptor, LGI1, CASPR2, amphiphysin, PNMA2 (Ma2/Ta), Hu, Ri, CV2, Yo, SOX1, Tr (DNER), Zic4, titin, and recoverin. Myositis antibodies and repetitive nerve stimulation (RNS) were performed when myopathy or neuromuscular junction disorders were suspected.

### Neuroimaging

All patients underwent brain and orbital MRI, including T1-weighted, T2-weighted, fluid-attenuated inversion recovery (FLAIR), and diffusion-weighted imaging (DWI) sequences. Gadolinium-enhanced T1-weighted imaging was performed when clinically appropriate. Two patients additionally underwent whole-body positron emission tomography/computed tomography (PET-CT) to screen for underlying malignancy. Neuroimaging findings were reviewed by two experienced neuroradiologists blinded to clinical outcomes.

### Video-oculography (VOG)

VOG was performed using VisualEyes system (Interacoustics, Denmark).VOG was performed in one patient at initial presentation and during relapse to document longitudinal ocular motor changes. Measurements included spontaneous nystagmus, saccadic velocity, and horizontal and vertical pursuit gain. All recordings were obtained using standardized protocols and analyzed by a neuro-ophthalmologist with expertise in ocular motor physiology.Vertical saccade velocities were extracted from bilateral channels (left and right) under each condition. Multiple measurements obtained within each session were pooled to generate a representative value. Data are presented descriptively as mean ± standard deviation (SD), based on repeated measurements rather than independent samples. Individual data points are also displayed to illustrate measurement variability.Pursuit gain was defined as the ratio of eye velocity to target velocity.

Saccade velocity was measured in degrees per second (°/s), and compared with established normal values (> 200°/s for vertical saccades in healthy adults).

### Literature review

A systematic literature search was conducted using PubMed from January 1995 to December 2025. Search terms included: (“glutamic acid decarboxylase” OR “GAD” OR “GAD65”) AND (“nystagmus” OR “diplopia” OR “ptosis” OR “gaze” OR “ocular motor” OR “saccade” OR “flutter” OR “vestibular”). Reference lists of included publications were screened to identify additional eligible articles. Studies reporting ocular manifestations or vestibulo-ocular motor findings associated with anti-GAD antibody–related neurological syndromes were included. Non-English articles and duplicated reports were excluded.

## Results

### Clinical characteristics

The four patients (2 males, 2 females) had ages at onset ranging from 53 to 68 years. Time from symptom to diagnosis varied from 55 to 340 days(approximately 2–11 months)(detailed timelines are provided in Supplementary Table S1). Ocular symptoms were among the earliest clinical features in three patients (Patients 2–4); however, in one patient (Patient 4), the temporal relationship between the initial ocular symptom and the final diagnosis was uncertain. Patient 1 first developed gait instability, followed by diplopia six months later.

Patient 2: reported “objects jumping” during fixation and exhibited spontaneous downbeat nystagmus. At relapse two years later, the patient developed dizziness, gait instability, and limb ataxia.

Patient 3: presented with diplopia. Examination revealed partial left abduction limitation and gaze-evoked horizontal nystagmus. Eight months later, the patient relapsed with dizziness, gait instability, and memory decline, accompanied by spontaneous downbeat nystagmus and cerebellar ataxia. EEG(electroencephalogram) at relapse was abnormal. Cognitive assessments revealed progressive decline in MoCA scores(28).

Patient 4: initially presented with isolated ptosis, with positive bilateral eyelid fatigue testing. However, given the five-year interval before the development of systemic neurological symptoms, including limb weakness, dysphagia, and dyspnea, the disease onset was defined as the time when these characteristic neurological manifestations emerged. The relationship between the initial ptosis and the later diagnosis remains uncertain. Electromyography was abnormal and gastrocnemius muscle biopsy showed neurogenic alterations. RNS was normal (Table 1).

**Table 1:** Basic Clinical Characteristics of the Four Patients with Anti-GAD Antibody–Associated Neurological Syndromes Presenting with Ocular Manifestations

Patient	Sex	Age at onset (years)	Time to diagnosis (days)	Ocular manifestations	Other symptoms	Neurological signs	Anti-GAD65 antibody (Serum / CSF)	Brain MRI	PET-CT	CSF findings	Acute treatment	Immunosuppressant (maintenance)	Relapse	mRS at onset	mRS at follow-up
1	M	62	207	Diplopia	Dizziness; gait instability; dysarthria	Dysarthria; partial limitation of upward gaze; limb and gait ataxia	1:32 / 1:100	No specific abnormality	No specific abnormality	WBC: 0 × 10⁶/L; protein: 77 mg/dL; OCB (+)	Two courses of IVIG + corticosteroids	None	No	4	4
2	M	59	340	Nystagmus	Dizziness; gait instability	Spontaneous downbeat nystagmus; limb and gait ataxia	1:320 / 1:320	Normal initially; cerebellar atrophy on relapse	No specific abnormality	WBC: 4 × 10⁶/L; protein: 20.6 mg/dL	IVIG + corticosteroids	Mycophenolate mofetil → azathioprine	Yes	2	4
3	F	53	84	Diplopia	Dizziness; gait instability; memory decline	Partial limitation of left abduction; gaze-evoked nystagmus; spontaneous downbeat nystagmus; gait ataxia	1:100 / NA	No specific abnormality	Not performed	Not performed	IVIG + corticosteroids	Mycophenolate mofetil	Yes	1	2
4	F	68	55*	Ptosis	Limb weakness and myalgia; dysphagia; dyspnea	Ptosis; reduced limb muscle strength	1:320 / NA	No specific abnormality	Not performed	Not performed	Corticosteroids	Mycophenolate mofetil (discontinued after 3 months)	Yes	1	2

*Abbreviations*: *IVIG* intravenous immunoglobulin, *mRS* modified Rankin Scale, *OCB* oligoclonal bands, *WBC* white blood cells, *NA* not assessed

*For Patient 4: disease onset was defined as the time of systemic neurological symptom emergence rather than the initial isolated ptosis

### Laboratory findings

All four patients had positive serum anti-GAD65 antibodies.CSF anti-GAD65 antibodies were assessed in two patients (Patients 1 and 2), both of whom were positive. Lumbar puncture performed in Patients 1 and 2 revealed mildly elevated CSF protein and normal cells in Patient 1, and normal CSF studies in Patient 2. CSF oligoclonal bands were positive in Patient 1 and absent in Patient 2. Patient 3 was positive for anti-β2-glycoprotein I and antinuclear antibodies. Patient 4 showed markedly elevated creatine kinase and serum anti-titin antibody positivity and anti-thyroglobulin antibody positivity, while AChR/MuSK/RYR/LRP4 antibodies and myositis-specific antibodies were all negative (Table 1).

### Neuroimaging findings

**Fig. 1:**
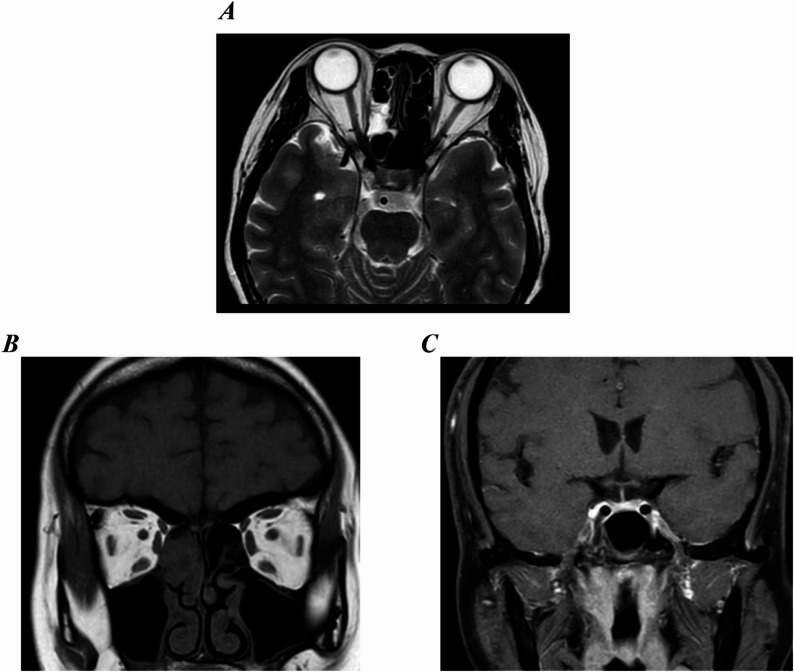
Neuroimaging of Patient 3. **A**: T2-weighted orbital MRI at initial presentation showing the left eye-globe in an adducted position. **B**: T1-weighted orbital MRI demonstrating normal extraocular muscles with no structural abnormalities. **C**: Gadolinium-enhanced T1-weighted MRI showing no abnormal enhancement of the cavernous sinus

**Fig. 2:**
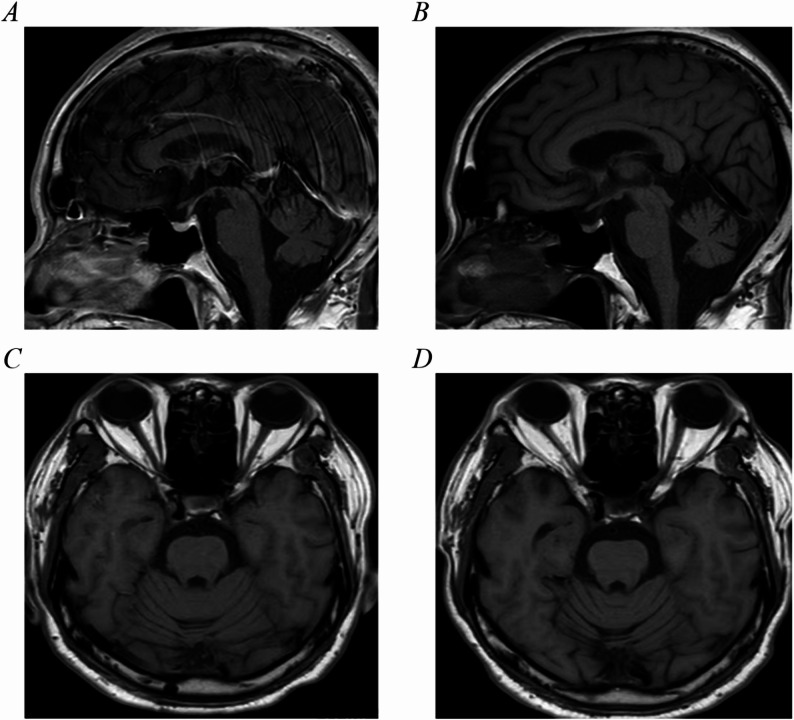
Neuroimaging of Patient 2. **A**: Sagittal T1-weighted MRI at initial presentation showing no structural abnormalities. **B**: Sagittal T1-weighted MRI at relapse revealing cerebellar atrophy. **C**: Axial T1-weighted MRI at initial presentation showing no abnormalities. **D**: Axial T1-weighted MRI at relapse demonstrating cerebellar atrophy

Initial brain and orbital MRI were unremarkable in all patients (Fig. 1). Patient 2 experienced relapse two years after diagnosis, at which time repeat brain MRI showed cerebellar atrophy (Fig. 2). Whole-body PET-CT in Patients 1 and 2 demonstrated no abnormal metabolic activity or evidence of neoplasm.

### Video-oculography (VOG) findings

**Fig. 3:**
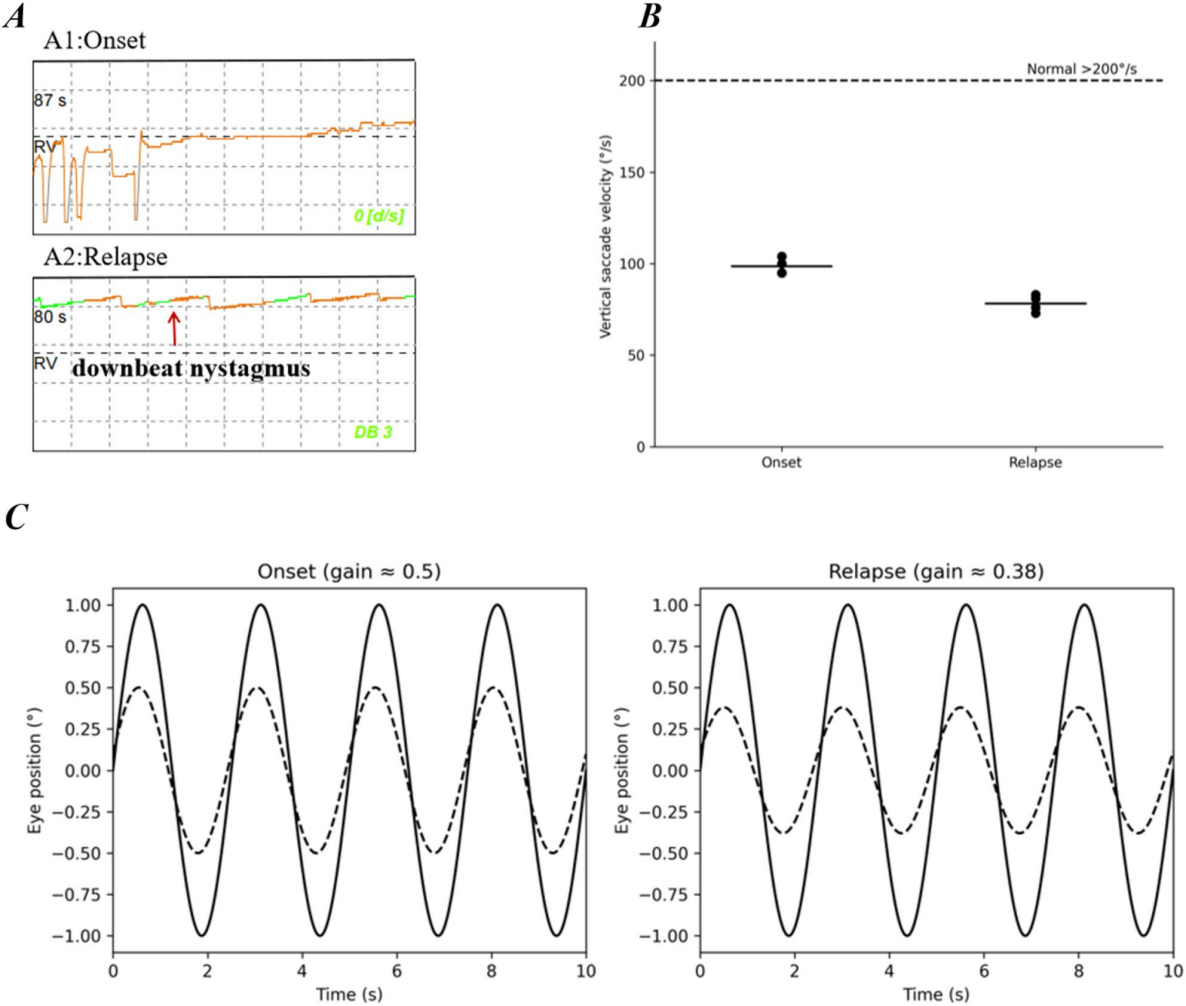
VOG of Patient 3. Velocity traces are expressed in degrees per second (°/s), and position traces in degrees (°). **A**: Spontaneous nystagmus recordings (vertical channels) in Patient 3: At onset(A1), no clear vertical nystagmus was observed. During relapse(A2), downbeat nystagmus emerged under no fixation conditions, indicating progressive cerebellar dysfunction. **B**: Scatter plot of vertical saccade velocities showing individual measurements. Horizontal lines indicate mean values. Vertical saccade velocity decreased from 98.5°/s at onset to 78.2°/s at relapse, suggesting progressive impairment of cerebellar ocular motor function (normal > 200°/s). **C**: Horizontal smooth pursuit recordings at 0.4 Hz. Solid lines represent target motion, and dashed lines represent eye position. At onset, eye movements closely followed the target with a gain of approximately 0.5. During relapse, pursuit gain decreased to approximately 0.38, with increased deviation between target and eye position, suggesting impaired cerebellar ocular motor control

Patient 3 VOG showed abnormal in vertical saccade and horizontal pursuit. Onset showed reduced vertical saccadic velocity and horizontal pursuit gain. During relapse, VOG demonstrated spontaneous downbeat nystagmus, markedly impaired vertical saccadic velocity, and worsened horizontal pursuit gain (Fig. 3).

### Treatment and outcomes

Cases 1–3 received high-dose intravenous corticosteroids combined with intravenous immunoglobulin(IVIG) in the acute phase; Case 1 received a second IVIG course after one month. Case 4 received high-dose corticosteroids alone. All four patients improved after initial treatment, and creatine kinase normalized in Case 4.

Patient-specific outcomes were as follows: Case 1: No maintenance immunotherapy; persistent diplopia and gait instability (mRS 4).Case 2: Relapsed after discontinuing steroids due to arthralgia; stabilized after IVIG and immunosuppressants, though dizziness and ataxia persisted (mRS 4).Case 3: Relapsed despite corticosteroids; stabilized after IVIG and mycophenolate mofetil, with residual dizziness and cognitive decline (mRS 2).Case 4: Improved initially; relapse with CK elevation after stopping mycophenolate mofetil (mRS 2) (Table 1).

### Literature review

A total of 67 articles were identified, of which 61 met the inclusion criteria. Reports included diplopia alone (*n* = 11), nystagmus alone (*n* = 27), both diplopia and nystagmus (*n* = 12), isolated ptosis (*n* = 2), and isolated gaze abnormalities (*n* = 3). The remaining six articles described mixed ocular presentations.

Seventeen studies provided detailed vestibulo-ocular motor characteristics. The most frequently reported abnormalities involved saccadic and pursuit impairments, particularly multistep saccades and square-wave oscillations. Three studies documented longitudinal VOG changes before and after treatment (Table 2).

**Table 2:** Summary of Reported Ocular and Vestibulo-Ocular Motor Features in Anti–GAD Antibody–Associated Neurological Syndromes

Reference	Number of cases	Ocular Manifestations	Vestibulo-Ocular Motor Features
Oskarsson et al. (2008)	1	Supranuclear vertical gaze palsy	Vertical saccade hypometria and prolonged latency; Vertical pursuits saccadic; Horizontal pursuits normal
Skiadopoulos (2024)	1	Vertical diplopia; Strabismus	Normal
Kodama et al. (2020)*	1	Diplopia; Upbeat nystagmus; Downbeat nystagmus	Slow saccades; Multi-component saccades
Thomas et al. (2005)*	1	Diplopia; Nystagmus; Strabismus; Bilateral mild abduction deficit	Horizontal and vertical slow saccades
Economides & Horton (2005)	1	Gaze-evoked nystagmus; Diplopia; Bilateral abduction deficit; Strabismus	Impaired horizontal pursuit; Impaired saccadic initiation; Vertical saccades and pursuits normal
Hac et al. (2023)	1	Downbeat nystagmus; Gaze-evoked nystagmus with centripetal and rebound nystagmus	Horizontal and vertical slow saccades
Theeranaew et al. (2022)	10	Downbeat nystagmus; Upbeat nystagmus; gaze-evoked nystagmus	Saccadic intrusions
Mahale et al. (2021)	1	Upbeat nystagmus	Saccades and pursuit normal
Feldman et al. (2019)	1	Upbeat nystagmus; Gaze-evoked nystagmus	Hypometric saccades; Catch-up saccades
Baizabal-Carvallo & Alonso-Juarez (2018)	1	Downbeat nystagmus	Hypermetric saccades
Shaikh & Wilmot (2016)	1	Ocular flutter; Downbeat nystagmus	Impaired horizontal and vertical pursuit
Brokalaki et al. (2015)*	1	Gaze-evoked nystagmus; Downbeat nystagmus	Square-wave oscillations
Zivotofsky et al. (2006)	1	Downbeat nystagmus	Prolonged saccadic latency; Impaired downward pursuit; Multi-component saccades
Tilikete et al. (2005)	2	Periodic alternating nystagmus; Gaze-evoked nystagmus; Downbeat nystagmus	Abnormal pursuit; Slowed vertical saccades
Honnorat et al. (1995)	1	Horizontal rotary nystagmus	Upward saccades absent; Downward saccades preserved; Multi-component saccades
Wang et al. (2021)	8	Diplopia; Alternating skew deviation; Abduction deficit; Downbeat nystagmus; Gaze-evoked nystagmus	Saccadic pursuit; Saccadic abnormalities
Muñiz-Castrillo et al. (2020)	1	Diplopia; Gaze-evoked nystagmus; Downbeat nystagmus	Impaired pursuit

Studies are cited using author–year format to avoid numbering conflicts with the main reference list

Full bibliographic details are provided in Supplementary Materials

*Studies marked with an asterisk provided longitudinal comparisons before and after treatment

## Discussion

In this study, we describe four patients with anti–GAD65 antibody–associated neurological syndromes presenting with prominent oculomotor abnormalities, and integrate these findings with a comprehensive literature review. Our results confirm that diplopia, nystagmus, and ptosis are common clinical manifestations and may occur early in the disease course and, in some cases, precede broader neurological involvement. Importantly, we extend previous observations by providing longitudinal quantitative video-oculography (VOG) data and by proposing a structured framework for classifying oculomotor phenotypes.

### Oculomotor manifestations as early indicators of anti-GAD65–associated disease

Consistent with prior reports, ocular motor abnormalities were among the earliest manifestations in the majority of our patients. Notably, downbeat nystagmus was observed in two cases and emerged during disease evolution in one patient. This finding is in line with previous study [12] linking downbeat nystagmus to dysfunction of the cerebellar flocculus–paraflocculus complex, a region critically involved in vertical gaze stabilization. The appearance of downbeat nystagmus in the absence of early MRI abnormalities further supports the concept that functional impairment of cerebellar circuits may precede structural changes.Diplopia and gaze limitation observed in our cohort likely reflect heterogeneous mechanisms, including involvement of brainstem ocular motor nuclei, cerebellar modulation of eye movements, and possibly immune-mediated effects on extraocular muscles [10, 15–17].This heterogeneity underscores the diagnostic challenge of anti-GAD65–associated syndromes when presenting with isolated oculomotor symptoms.

### A structured oculomotor phenotype framework

Although individual ocular motor abnormalities in anti-GAD65–associated disorders have been well described, previous studies have largely reported these findings in isolation without systematic phenotypic integration. Based on our cases and literature synthesis, we propose a structured oculomotor phenotype framework comprising three patterns: (1) Cerebellar pattern, characterized by downbeat nystagmus, impaired smooth pursuit, and saccadic dysmetria or slowing; (2) Brainstem-related pattern, including gaze palsy, diplopia, and gaze-evoked nystagmus; (3) Neuromuscular-like pattern, presenting with fatigable ptosis or muscle involvement. This framework may help clinicians interpret seemingly disparate ocular findings within a unified pathophysiological context and facilitate earlier recognition of autoimmune etiologies. To our knowledge, such a pattern-based classification integrating clinical observations with vestibulo-ocular motor features has not been previously formalized.

### Longitudinal VOG as a tool for dynamic disease monitoring

A key strength of our study is the incorporation of longitudinal VOG data, which provides objective and quantitative assessment of ocular motor function. In Patient 3, we observed progressive slowing of vertical saccadic velocity and declining pursuit gain during relapse, accompanied by the emergence of vertical nystagmus. These findings are consistent with evolving cerebellar dysfunction and parallel clinical deterioration. Previous studies have described ocular motor abnormalities in anti-GAD65–associated disorders; however, longitudinal quantitative data remain scarce [18–20].Our results suggest that VOG may detect subtle functional changes that precede or accompany clinical relapse, thereby serving as a potential biomarker for disease activity. This is particularly relevant in cases where conventional neuroimaging remains normal in early stages.

### Atypical features and diagnostic considerations

We also identified anti-titin antibody positivity with elevated creatine kinase in one patient, suggesting possible muscle involvement [21].In this case, ptosis preceded the development of systemic neurological manifestations by several years. However, given this prolonged temporal interval, the early ocular symptom cannot be confidently attributed to the same disease process. The temporal dissociation raises the possibility that the ptosis was either unrelated or represented a nonspecific or prodromal manifestation.Therefore, a direct pathogenic association cannot be established, and the observed sequence should be interpreted with caution.This case nevertheless highlights the complexity of clinical presentations and underscores the importance of careful longitudinal evaluation in patients with atypical or evolving features, rather than providing definitive evidence of a shared autoimmune mechanism.

### Clinical implications

The recognition of oculomotor abnormalities as early manifestations of anti-GAD65–associated neurological syndromes has important clinical implications. Patients presenting with unexplained diplopia, nystagmus, or ptosis—especially in the absence of structural abnormalities on MRI—should prompt consideration of autoimmune etiologies, including anti-GAD65–associated disease. Early identification may facilitate timely initiation of immunotherapy, which is critical given the potential for relapse and progression. The development of cerebellar atrophy in one of our patients highlights the risk of irreversible structural damage if treatment is delayed or insufficient. In this context, longitudinal VOG assessment may provide an additional tool for monitoring treatment response and guiding immunosuppressive strategies.

## Limitations

This study has several limitations. First, the sample size is small, reflecting the rarity of the condition, and limits generalizability. Second, the retrospective design introduces potential selection and information biases. Third, VOG data were available in only one patient, precluding systematic analysis across cases. In addition, CSF and electrophysiological data were not uniformly available. These limitations should be considered when interpreting our findings.

## Future directions

Future studies with larger cohorts and prospective designs are needed to validate the proposed oculomotor phenotype framework and to determine the diagnostic and prognostic value of specific ocular motor abnormalities. Standardized VOG protocols may further clarify the temporal relationship between functional impairment, clinical relapse, and structural changes on neuroimaging.

## Conclusions

In conclusion, oculomotor abnormalities may represent early clinical features of anti-GAD65 antibody–associated neurological syndromes. Downbeat nystagmus may serve as a functional marker of cerebellar involvement, potentially preceding structural imaging changes. Longitudinal VOG provides objective insights into disease dynamics and may complement clinical and radiological assessment. The proposed oculomotor phenotype framework offers a structured approach to interpreting ocular findings and may facilitate earlier diagnosis and improved disease monitoring.

## Supplementary Information

Supplementary Material 1.

Supplementary Material 2.

Supplementary Material 3.

## Data Availability

The datasets generated and/or analyzed during the current study are available from the corresponding author on reasonable request.

## Abbreviations

GAD: Glutamic acid decarboxylase
VOG: Video-oculography
MRI: Magnetic resonance imaging
CK: Creatine kinase
mRS: Modified Rankin Scale
CSF: Cerebrospinal fluid
NMDA: N-Methyl D-Aspartate
AMPA: Aminomethylphosphonic acid
GABAB: Gamma-aminobutyric acid B
LGI1: Leucine-rich, glioma inactivated 1
CASPR2: Contactin-associated protein-like 2
RNS: Repetitive nerve stimulation
FLAIR: Fluid-attenuated inversion recovery
DWI: Diffusion-weighted imaging
PET-CT: Positron emission tomography/computed tomography
EEG: Electroencephalogram
MoCA: Montreal cognitive assessment
MMSE: Mini-mental state examination
AChR: Acetylcholine receptor
MuSK: Muscle skeletal receptor tyrosine kinase
RYR: Ryanodine receptor
LRP4: Low density lipoprotein receptor related protein 4
IVIG: Intravenous immunoglobulin
