# Supplementary material for: Oculomotor phenotypes and longitudinal video-oculography dynamics in anti-GAD65 antibody–associated neurological syndromes: a case series and literature review
Source: BMC Neurol. 2026 Jun 11;26:514. doi: 10.1186/s12883-026-05014-0 (PMC13474773; doi:10.1186/s12883-026-05014-0)
Supplement: Supplementary file 1 [file 12883_2026_5014_MOESM2_ESM.pdf]

MoCA
COGNITIVE ASSESSMENT

Version 8.1 English

Name:
Education: Date of birth:
Sex: DATE:

**VISUOSPATIAL / EXECUTIVE**			POINTS

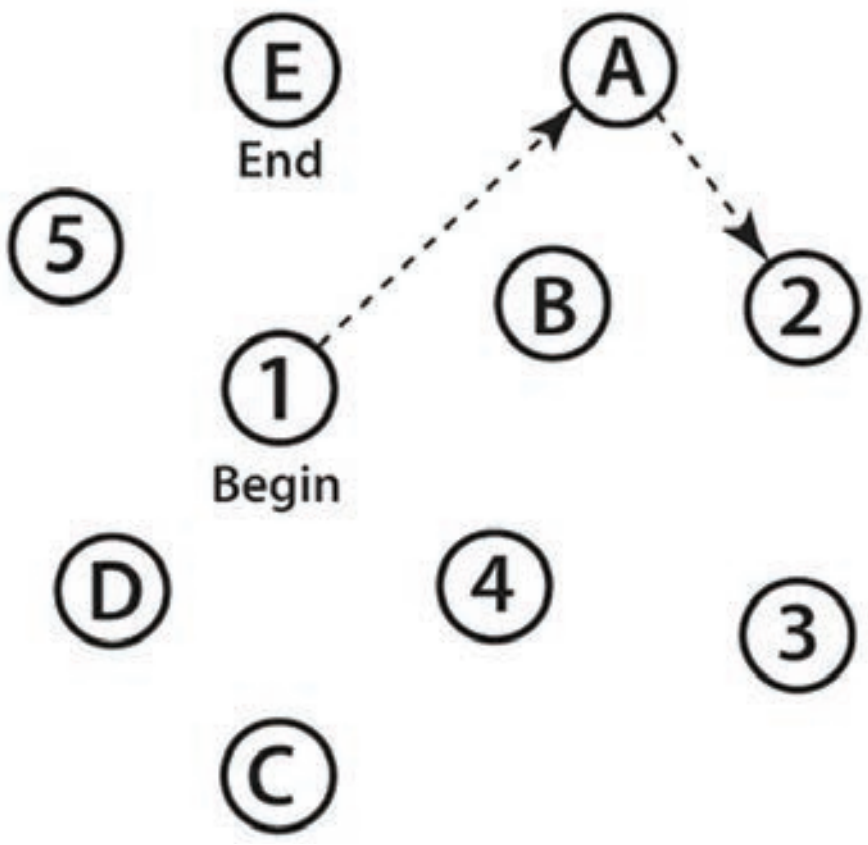

[ ]

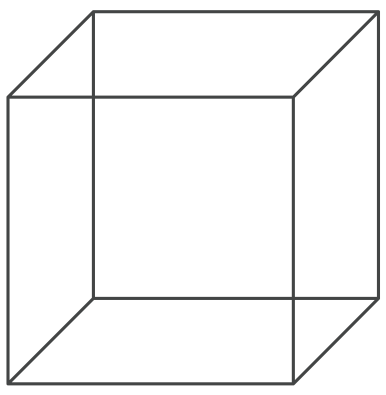

Copy cube

[ ]

Draw CLOCK (Ten past eleven)
(3 points)

[ ] Contour [ ] Numbers [ ] Hands

___ /5

NAMING

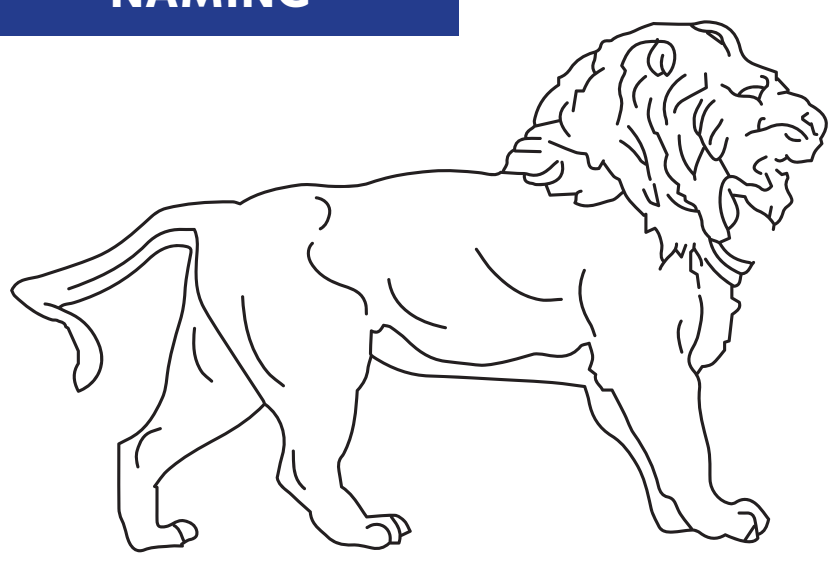

[ ]

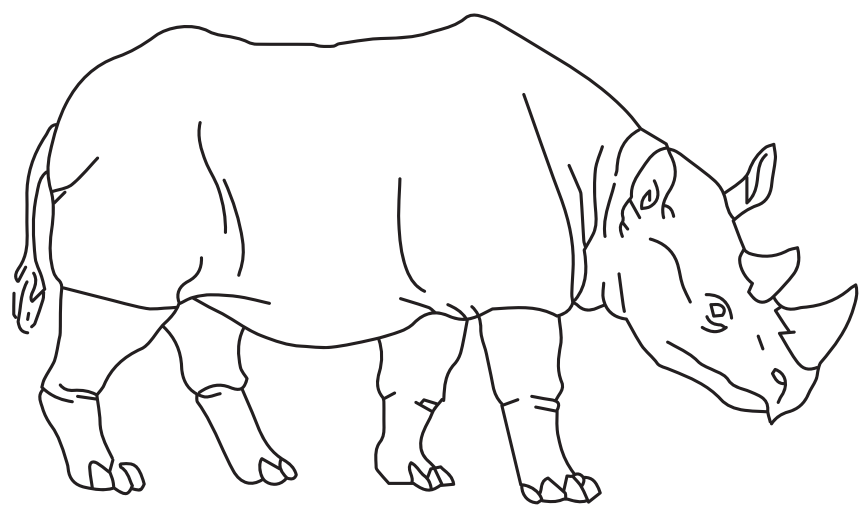

[ ]

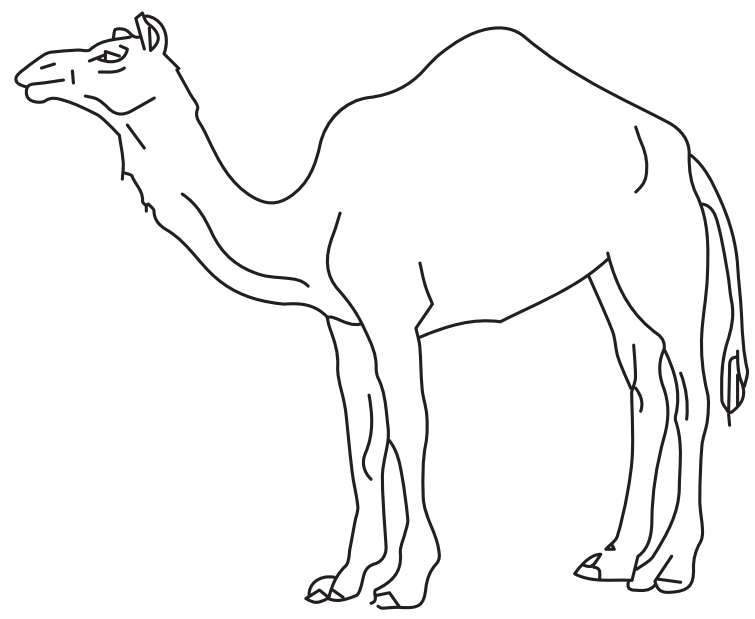

[ ]

___ /3

MEMORY

Read list of words, subject must repeat them. Do 2 trials, even if 1st trial is successful. Do a recall after 5 minutes.

	FACE	VELVET	CHURCH	DAISY	RED
1ST TRIAL					
2ND TRIAL					

NO POINTS

ATTENTION

Read list of digits (1 digit/ sec.).

Subject has to repeat them in the forward order. [ ] 2 1 8 5 4

Subject has to repeat them in the backward order. [ ] 7 4 2

___ /2

Read list of letters. The subject must tap with his hand at each letter A. No points if ≥ 2 errors

[ ] F B A C M N A A J K L B A F A K D E A A A J A M O F A A B

___ /1

Serial 7 subtraction starting at 100. [ ] 93 [ ] 86 [ ] 79 [ ] 72 [ ] 65

4 or 5 correct subtractions: **3 pts**, 2 or 3 correct: **2 pts**, 1 correct: **1 pt**, 0 correct: **0**

___ /3

LANGUAGE

Repeat: I only know that John is the one to help today. [ ]
The cat always hid under the couch when dogs were in the room. [ ]

___ /2

Fluency: Name maximum number of words in one minute that begin with the letter F. [ ] _____ (N ≥ 11 words)

___ /1

ABSTRACTION

Similarity between e.g. orange - banana = fruit [ ] train - bicycle [ ] watch - ruler

___ /2

DELAYED RECALL

(MIS)		FACE	VELVET	CHURCH	DAISY	RED	
	Has to recall words WITH NO CUE	[ ]	[ ]	[ ]	[ ]	[ ]	Points for UNCUED recall only
X3							
X2	Category cue						MIS = ____ /15
X1	Multiple choice cue						

Memory Index Score (MIS)

___ /5

ORIENTATION

[ ] Date [ ] Month [ ] Year [ ] Day [ ] Place [ ] City

___ /6

© MoCA Test Inc.
Created by Z. Nasreddine MD

www.mocacognition.com

Training and Certification are required to ensure accuracy.

MIS: /15
(Normal ≥ 26/30)
Add 1 point if ≤ 12 yr edu

TOTAL ___ /30

ADMINISTERED BY Dai, Feifei NOT MOCA CERTIFIED
