# Supplementary material for: Oculomotor phenotypes and longitudinal video-oculography dynamics in anti-GAD65 antibody–associated neurological syndromes: a case series and literature review
Source: BMC Neurol. 2026 Jun 11;26:514. doi: 10.1186/s12883-026-05014-0 (PMC13474773; doi:10.1186/s12883-026-05014-0)
Supplement: Supplementary file 2 [file 12883_2026_5014_MOESM1_ESM.docx]

Oskarsson B, Pelak V, Quan D, Hall D, Foster C, Galetta S. Stiff eyes in stiff-person syndrome. Neurology. 2008;71(5):378-380.

Skiadopoulos D. Diplopia management in a patient with stiff person syndrome. Clin Exp Optom. 2024;107(4):473-474.

Kodama S, Tokushige S, Sugiyama Y, Sato K, Otsuka J, Shirota Y, Hamada M, Iwata A, Toda T, Tsuji S, Terao Y. Rituximab improves not only back stiffness but also "stiff eyes" in stiff person syndrome: Implications for immune-mediated treatment. J Neurol Sci. 2020;408:116506.

Thomas S, Critchley P, Lawden M, Farooq S, Thomas A, Proudlock FA, Constantinescu CS, Gottlob I. Stiff person syndrome with eye movement abnormality, myasthenia gravis, and thymoma. J Neurol Neurosurg Psychiatry. 2005;76(1):141-142.

Economides JR, Horton JC. Eye movement abnormalities in stiff person syndrome. Neurology. 2005;65(9):1462-1464.

Hac NEF, Murphy OC, Butala AA, Newsome SD, Gold DR. Centripetal Nystagmus, Slow Saccades, Cerebellar Ataxia, and Parkinsonism in a Patient With Anti-GAD65-Associated Stiff Person Syndrome Spectrum Disorder. J Neuroophthalmol. 2023;43(2):273-276.

Theeranaew W, Wang F, Ghasia FF, Wilmot G, Shaikh AG. Gaze-holding and anti-GAD antibody: prototypic heterogeneous motor dysfunction in immune disease. Cerebellum. 2022;21(1):55-63.

Baizabal-Carvallo JF, Alonso-Juarez M. Vertical nystagmus associated with glutamic acid decarboxylase antibodies responding to cyclophosphamide. J Neuroimmunol. 2018;317:5-7.

Shaikh AG, Wilmot G. Opsoclonus in a patient with increased titers of anti-GAD antibody provides proof for the conductance-based model of saccadic oscillations. J Neurol Sci. 2016;362:169-173.

Brokalaki C, Kararizou E, Dimitrakopoulos A, Evdokimidis I, Anagnostou E. Square-Wave Ocular Oscillation and Ataxia in an Anti-GAD-Positive Individual With Hypothyroidism. J Neuroophthalmol. 2015;35(4):390-395.

Zivotofsky AZ, Siman-Tov T, Gadoth N, Gordon CR. A rare saccade velocity profile in Stiff-Person Syndrome with cerebellar degeneration. Brain Res. 2006;1093(1):135-140.

Honnorat J, Trouillas P, Thivolet C, Aguera M, Belin MF. Autoantibodies to glutamate decarboxylase in a patient with cerebellar cortical atrophy, peripheral neuropathy, and slow eye movements. Arch Neurol. 1995;52(5):462-468.

Muñiz-Castrillo S, Vogrig A, Joubert B, Pinto A-L, Gonçalves D, Chaumont H, Rogemond V, Picard G, Fabien N, Honnorat J.Transient Neurological Symptoms Preceding Cerebellar Ataxia with Glutamic Acid Decarboxylase Antibodies. Cerebellum. 2020;19(5):715-721.
