# Supplementary material for: Oculomotor phenotypes and longitudinal video-oculography dynamics in anti-GAD65 antibody–associated neurological syndromes: a case series and literature review
Source: BMC Neurol. 2026 Jun 11;26:514. doi: 10.1186/s12883-026-05014-0 (PMC13474773; doi:10.1186/s12883-026-05014-0)
Supplement: Supplementary file 3 [file 12883_2026_5014_MOESM3_ESM.docx]

TableS1:Clinical Timeline of Each Patient

| Patient | Day Onset | Day Neuroimaging | Day Laboratory Testing | Day Treatment |
| --- | --- | --- | --- | --- |
| 1 | 0 | 186 | 206 | 207 |
| 2 | 0 | 330 | 331 | 340 |
| 3 | 0 | 65 | 67 | 84 |
| 4 | 0 | 47 | 48 | 55 |
